# Cross-Sectional Imaging to Evaluate the Risk of Rupture in Abdominal Aortic Aneurysms: Review article based on a dissertation submitted to fulfill the academic grade of doctor in medical sciences (….), entitled: Imaging the mechanisms involved in abdominal aortic aneurysms rupture; a step towards patient-specific risk assessment.

**DOI:** 10.5334/jbr-btr.1204

**Published:** 2016-11-19

**Authors:** Alain Nchimi

**Affiliations:** 1 University of Liège, Belgium, BE

**Keywords:** Aneurysm, AAA, MRI, FDG, PET

## Introduction

In developed countries, abdominal aortic aneurysm (AAA) rupture represents an important cause of mortality, which is potentially avoidable [1]. In the current clinical recommendations, only the maximal aneurysmal diameter and its derivatives, such as growth rate, are significantly associated with risk of rupture on a population basis [(2,3]. Because AAAs with maximal diameter under the threshold do rupture [4,5] – even under surveillance [6] – whereas those above don’t always [7,8], the sensitivity and specificity of the diameter-based management scheme of patients with AAA are poor and raise patient-specific concerns, as they imply that an “acceptable” range of patients are either undertreated or overtreated. A potential change of this paradigm can be contemplated via patient-specific approaches to AAA rupture risk assessment afforded by new imaging concepts.

AAA rupture occurs when the internal force (stress) exceeds the wall strength. This biomechanical view has driven efforts to experimentally determine the failure properties of aortic tissue fragments and to evaluate the wall stress in vivo, computing finite element simulations (FES) based on tridimensional imaging data. However, the following understatement applies: “before the aneurysm is ruptured, it is not”, implying more complex models to determine AAA behavior. These models may in turn summarize AAA rupture as the end-stage loss-of-balance process between destructive and healing biological mechanisms. Therefore, this article questions the following: (i) How are biological processes in AAA evaluated and quantified from medical imaging? (ii) How do such derived image-based parameters correlate to parameters that neglect biological information, such as AAA diameter, wall stress, or patient outcomes?

## Rationale for a Patient-specific Assessment of AAA

Aortic aneurysm prevalence increases with age and reaches 5–10 percent of the population over 65 years of age [9]. AAA is a slow, pathological development that is mostly asymptomatic until possible rupture, its most common complication. Less frequent complications include periadventitial inflammation, thrombosis, and peripheral embolization. Rupture-related symptoms may be serious and lead to death before admission in nearly half of the patients and in another half of those operated on [10].

The natural history of aortic aneurysms is incompletely elucidated for several reasons. The first is the hypothetical nature of the events happening before diagnosis. Second, the hypothetical risk of rupture of larger aneurysms is the motivation for preventive interventions. Finally, many co-morbidity factors are found in patients with aortic aneurysm. The annual rate of aneurysmal rupture in people refusing surgery is estimated at 8 percent, 10 percent, and 20 percent, respectively, for aneurysms with maximum diameters of 55–59 mm, 60–69 mm, and greater than 70 mm [11].

Additionally, several studies have shown that the annual risk of AAA rupture statistically exceeds operative risk at the critical diameter of 55 mm in men and 50 mm in women [2,3,12,13,14]. The initial diameter of AAA is therefore a critical parameter for risk assessment, but the risk of rupture of AAA is mainly determined by its growth rate in clinical practice [15], as the diameter is only a weak factor of growth [16,17,18]. In fact, due to several interplaying factors, the growth curve of AAA with time is not linear and may have exponential, quadratic, or plateau phases [15,19]. The question of whether AAA expansion rate and rupture risk are actually related remains open, but growth rate acceleration clearly indicates a deleterious change of the biomechanical status of the aneurysm, suggesting that anticipation or early detection may be of value to prevent eventual rupture.

## Overview of the Mechanisms Involved in AAA

It is generally accepted that both genetic and environmental risk factors are associated with the genesis of AAA through a common pathway that causes proteolytic depletion of the extracellular matrix (ECM), resulting in a structural imbalance [20]. Since the seminal works of Busuttil et al. [21] and Dobrin et al. [22] highlighting the implication of proteolysis of the ECM in the expansion and rupture of AAA, there has been a considerable advance in knowledge on proteolytic enzymes types and subtypes (mainly matrix metalloproteases (MMPs)), as well as their production and regulatory mechanisms [23,24,25,26,27,28,29]. Several mechanisms upregulate the ECM remodeling, including inflammatory and immune cell activities in response to pro-angiogenic cytokines [30,31,32,33]; excess oxidative stress [34,35], due, for example, to female gender and a genetic susceptibility for AAA rupture [36,37,38,39,40]; tobacco smoking [41]; hemagglutination (iron-mediated oxidation); immune dysfunction [42]; and infection [43,44,45,46].

Wall stress excess in AAA causes hypertrophic responses, resulting in vascular enlargement, elongation, and change of shape [47,48]. In addition to normal stress in the wall, shear stress, especially from blood flow, has implications in aneurysm development and progress. A study of amputated war survivors provides a nice illustration of the fact that wall shear stress may be a major player in AAA remodeling. Vollmar et al. studied 329 men who had lost a leg in World War II and 702 war veterans [49]. They observed AAA in 5.8 percent of the amputees compared with 1.1 percent of the non-amputees and concluded that “unilateral flow reduction after leg amputation causes an asymmetrical flow pattern at the aortic bifurcation, and this is probably the main cause of late damage to the aorta”. In addition, separated regions of rotational flow known as vortical structures may promote platelet activation and apposition of an intraluminal thrombus (ILT) [50,51], which is present in about 75 percent of all AAA [52,53]. The biomechanical effect of ILT is protective; that is, ILT carries some mechanical stress and buffers the wall from stress [54]. Unfortunately, ILT triggers a considerable number of biological activities [24,55,56,57,58,59,60], potentially deleterious to the wall strength and aneurysm outcomes [48,52,61,62].

On the other hand, some mechanisms protect the AAA wall from degradation, among which ECM fibrosis through fibroblast colonization and macromolecular crosslinking remains poorly investigated [63]. Calcification decreases elasticity and increases stiffness of the vessel wall [64] causing stress concentration, which in turn may explain the lower strength of the calcified AAA wall [65]. However, the calcified vessel wall is also thicker, such that the failure tension (strength × thickness) could almost remain unchanged when compared to the non-calcified, and thinner, vessel wall.

In summary, AAA results from imbalance between synthesis and destruction of the ECM as a consequence of polygenic predisposition and exposure to risk factors. Quiescence, or slow growth, of AAA indicates that the protective mechanisms tend to counterbalance those destroying the ECM. Its growth acceleration and rupture result from a breakdown of this equilibrium towards degradation and weakening in relation to an internal or an external trigger.

## General Principles of Imaging in AAA

### Conventional Imaging Techniques

AAA morphology can be evaluated by plain films, angiography, ultrasonography (US), computed tomography (CT), and magnetic resonance imaging (MRI). With the advent of cross-sectional techniques, plain films and angiography are being progressively abandoned because of their low information yield. US is harmless and, as such, useful in AAA screening programs. On the other hand, functional imaging is a growing concept that refers to the assessment of one or several pathophysiological pathways involved in AAA. As such, it includes tissue composition, metabolic, and molecular imaging.

#### Tissue Composition Imaging

Imaging techniques using X-rays have the ability to distinguish certain natural contrasts, such as calcium, water, fat, and air. However, tissue characterization by imaging became a refined concept only with the advent of MRI, owing to the wider range of the nuclear electromagnetic environment in human tissues. Some of the tissue components identifiable with imaging within the aortic wall and the underlying thrombus are potentially of interest in terms of risk stratification for AAA (Table 1).

**Table 1:** Imaging Properties of Components Involved in AAA.

Tissue of Interest	Imaging Properties				
	T1W MRI SI	T2W MRI SI	T2*W MRI SI	CT Attenuation	Enhancement*

Fat	High	High	High	Low	None
Acute hemorrhage (Iron)	Variable	Low	Low	High	None
Calcium	None	None	Low	Very High	None
Inflammation (edema, neovascularization)	Low	High	NA	Low	Yes

* Denotes the tissue properties after intravenous contrast agent administration.

#### Calcifications

**Figure 1:**
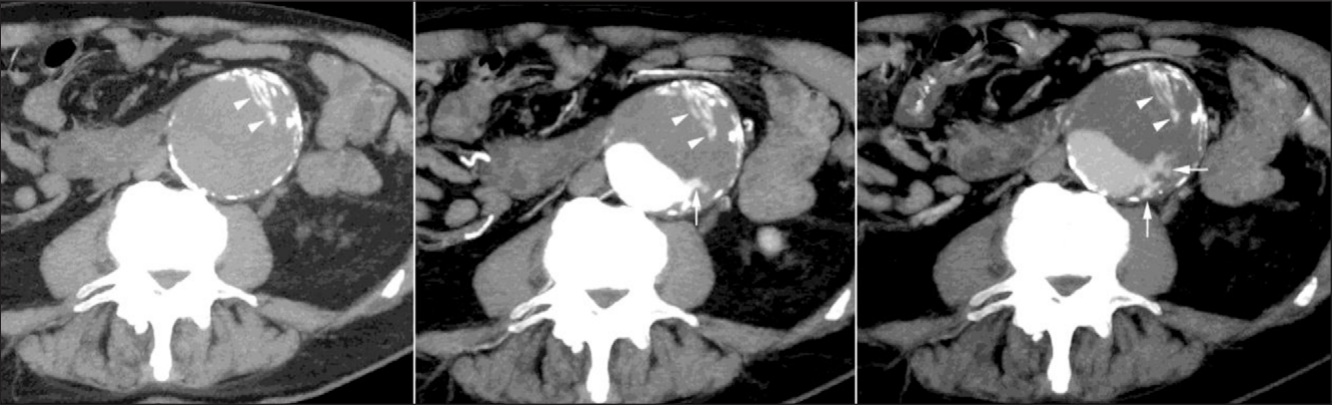
Transverse maximum intensity projection thick-slabs CT of a large AAA obtained before (left panel) and on arterial (centre panel) and portal (right panel) phases after intravenous injection of iodine contrast agent, showing both calcifications (arrowheads) and progressively enhancing vascular channels inside ILT (arrows).

**Figure 2:**
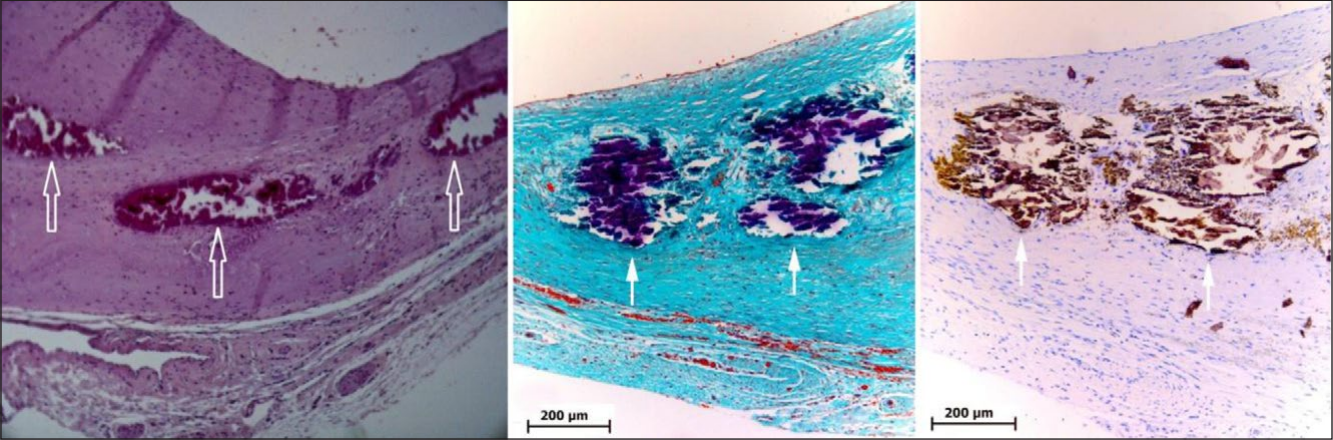
Hematoxylin-eosin staining (left panel) section of the aortic wall in a model of AAA by infusion of elastase in the rat, showing channels containing RBC within the ILT at early stages (open arrow). On older aneurysms, Masson trichrome and Von Kossa staining (resp. centre and right panels) show clusters of calcifications on similar locations (arrows).

Calcifications are easily detectable on X-ray–based imaging techniques (plain films, CT). Those located within ILT are of special interest because the calcification mechanism involving perivascular deposit implies that intra-thrombus former or current vascular channels should be present, both macroscopically (Figure 1) and microscopically (Figure 2). This unreported finding provides a potential explanation to how factors located deeper in the ILT can be exposed to the circulating blood cells.

#### Fat

**Figure 3:**
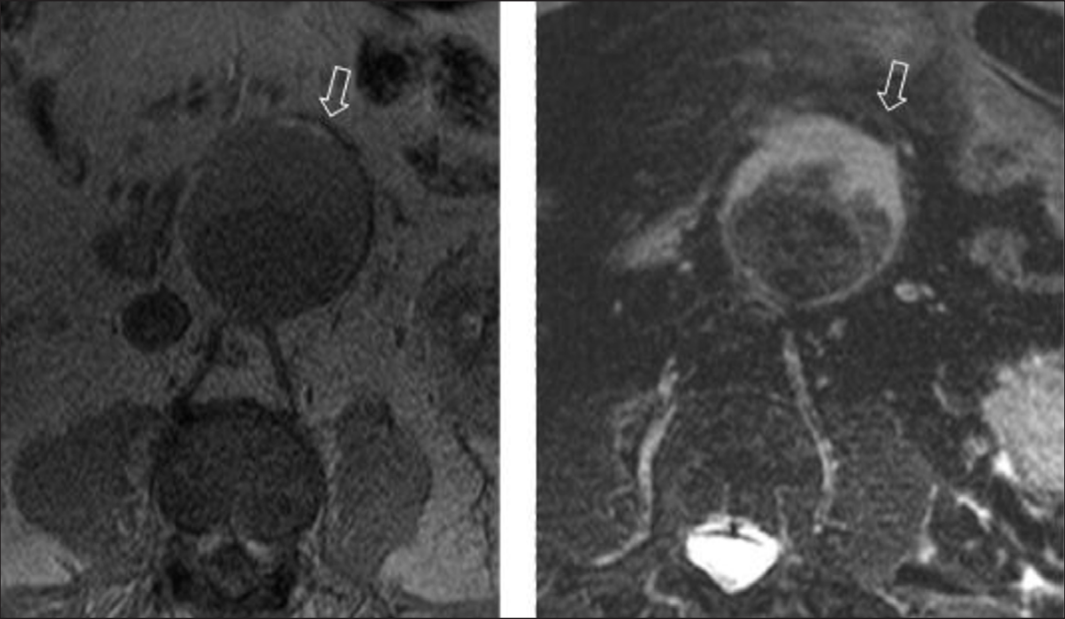
Transverse MRI of a large but stable AAA using a T1-weighted (left panel) and a T2-weighted image with fat saturation (right panel) in which the fat appears with high and very low signal, respectively (open arrows).

Fatty replacement (lipomatous metaplasia) is a rare and unreported feature of AAA. It may represent a cause of biomechanical weakening of the wall [66] but is mainly a marker of stabilization, as in other cardiovascular disease [67], although it has not been specifically evaluated so far. Fat is detected as low-attenuation tissue relative to water on X-ray techniques. It has a high signal on T1- and T2-weighted MRI. The decrease of its signal on MRI after a pulse or spectral fat suppression further helps fat identification (Figure 3).

#### Hemorrhage

**Figure 4:**
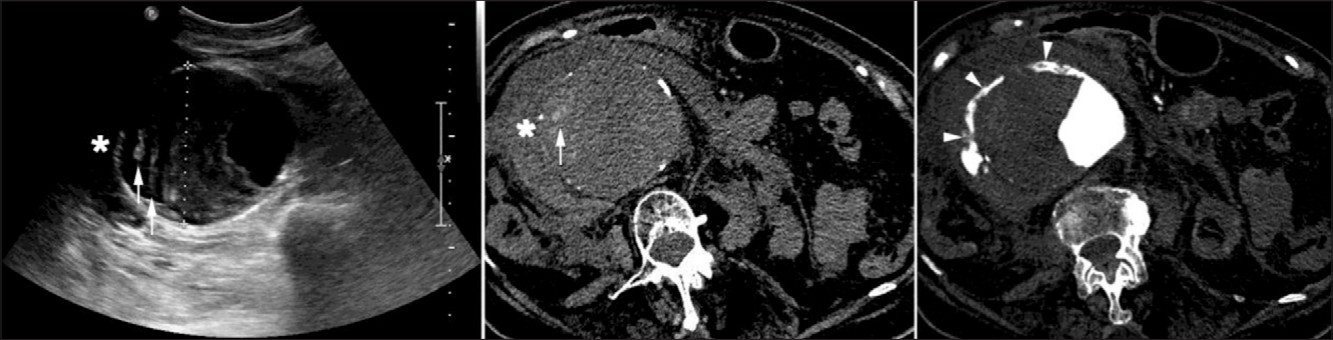
Transverse ultrasound (left panel) in an 89-year-old female admitted with shock showed a 11 cm AAA with a heterogeneous ILT, exhibiting an external hypoechoic crescent containing thick hyperechoic lines (arrows). Another hypoechoic structure surrounds the aorta (asterisk), suggesting a retroperitoneal hematoma. These intra-thrombus and retroperitoneal hematomas are confirmed by high attenuation (50–70 Hounsfield units) on unenhanced CT (centre panel). Contrast-enhanced CT (right panel) shows luminal contrast leaking into the periaortic hematoma through a channel dissecting the external ILT (arrowheads).

**Figure 5:**
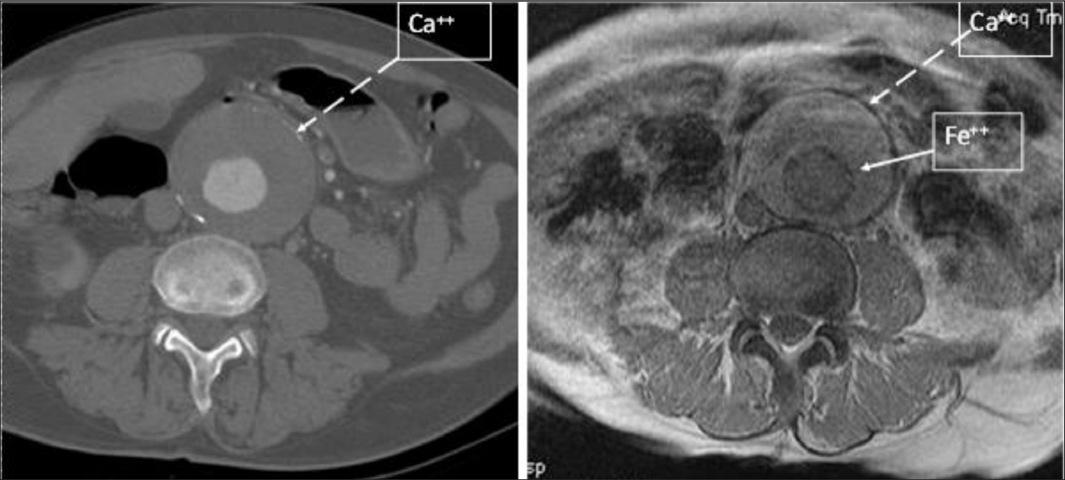
Transverse T2*-weighted MRI (right panel) of a large AAA showing abluminal and subadventitial low-signal rings. The corresponding contrast-enhanced CT transverse slice (left panel) shows subadventitial hyperdense calcifications (Ca++) but fails to replicate this finding around the aortic lumen, as CT is less sensitive to low iron (Fe++) concentrations than T2*-weighted MRI.

Emergency aneurysm repair is advised when acute hemorrhage within ILT or the surrounding tissues is detected, especially in association with clinical symptoms. The classic sign of acute hemorrhage is a semi-lunar high-attenuation layer within ILT on CT – the so-called “crescent sign” [68,69] (Figure 4). Nevertheless, AAA symptoms are not necessarily correlated with histological abnormalities [70]. It is not uncommon to find chronic foci of hemorrhage inside thrombus. On MRI, the signal of hemorrhage is influenced by its composition, and thus its age. A simplistic but reasonably accurate approach is to consider that the high signal intensity on T1-weighted MRI is associated with the presence of methemoglobin (intra- or extra-cellular) [71]. In foci of chronic hemorrhage, a low T2*-weighted signal indicates the presence of hemosiderin, whatever the T1-weighted signal. Of note, the low T2*-weighted SI caused by hemosiderin is virtually indistinguishable from that caused by calcification (Figure 5).

A single or repeated hemorrhage within ILT is biologically pejorative. Indeed, iron released by lysed RBC induces oxidation through the Fenton reaction [72], resulting, amongst others, in proteolysis and increased platelet aggregation [24,55,56,57,58,59].

### Metabolic Imaging – Molecular Imaging

#### Radionuclide Imaging

Single photon emission computed tomography (SPECT) is designed to evaluate the internal distribution of a radionuclide using a photon-sensitive camera rotating around the patient. Inversely, positron emission tomography (PET) produces images from an internal source by detecting the two opposite (coincident) photons resulting from positron annihilation after disintegration of a radionuclide. The first human whole-body PET was commercialized in 1978 and the first hybrid PET-CT and PET-MRI scanners in 2000 and 2011, respectively. Improved spatial resolution down to 4–7 mm, availability of cyclotrons [73], and radio-pharmaceuticals suited to this type of imaging [74] allowed PET and its variants to be established as cornerstones of molecular imaging.

Fluorodeoxyglucose (FDG) is one of the most popular radionuclides. Similar to glucose, FDG enters cells using the same membrane transporter (GLUT) and undergoes phosphorylation by hexokinase to get metabolized as FDG-6-phosphate. As the latter cannot enter glycolysis (the cycle of hexoses), it accumulates in cells [75]. As such, FDG is a glucose analog, and its accumulation identifies sites of increased uptake (glycolysis). FDG uptake is quantifiable. Assuming a 1 g/ml constant body volumetric mass, the standardized uptake value (SUV) of a region-of-interest (ROI) relates the uptake in a given tissue to the total dose injected to the patient. SUV_ROI_ = [FDG uptake_ROI_ (MBq/g) × body weight (g)]/injected activity (MBq).

To compensate for background noise, SUV_ROI_ is often normalized by the vascular SUV or the liver SUV, which are sensitive to most input or output bias. Even so, use of SUV remains subject to bias, although it is the most common quantitative uptake descriptor in clinical practice [76]. Lastly, partial volume effects also have to be considered and, if necessary, corrected, especially when the objects’ size are less than two times the full width at half maximum resolution in x-y and z directors (Nyquist limit).

#### Diffusion–weighted MRI

The concept of molecular imaging also belongs to MRI, with sequences evaluating the movement of water molecules (diffusion) in tissues, the so-called DW-MRI [77]. In short, on DW-MRI, a regular magnetic field inhomogeneity (or gradient) is generated for a defined duration and intensity. It results into a shift of the proton precession within the magnetic field. After a few milliseconds, a second magnetic gradient with the same intensity and duration than the first is applied in the opposite direction. The intensity of the resulting magnetization (and hence of the signal (S)) equals the signal before application of the first gradient (S_0_) minus the intensity related to the spins that have moved off plane in between the two gradients. The relationship between these signals and the apparent diffusion coefficient is given by the equation S/S0 = exp (–*b* × ADC), where *b* is the diffusion factor, which depends on acquisition parameters (field strength, gradient duration, etc.) and ADC is the apparent diffusion coefficient. As water diffusion is a multidirectional process, ADC in all planes can be calculated by obtaining images with two or more *b*-values: ADC (x,y,z) = l*n* [S2 (x,y,z) / S1 (x,y,z)] / (*b*1 – *b*2). ADC is directly proportional to the diffusion within the milieu and therefore on the latter’s molecular and cellular density.

DW-MRI is an established technique for clinically important applications such as acute stroke, white matter tract assessment, diagnosis, and therapeutic responses in tumours and inflammatory diseases [78,79]. DW-MRI necessitates no ionizing radiation, and the actual causes of signaling differ from those of FDG-PET but are similarly sensitive to the cellular density. Furthermore, the composite nature of DW-MRI signaling makes it sensitive to perfusion effects at low *b*-values, or the intravoxel incoherent motion, that reflect other important biological processes, such as edema, and angiogenesis. Because of an inherently low vascular background signal, DW-MRI may theoretically supplant FDG-PET in the detection of cellular infiltrates. Despite these potential advantages, the DW-MRI findings have, to our knowledge, never been evaluated in aortic aneurysms.

On both PET and MRI, the development of contrast agents and tracers with organ or physiological process-specific affinity support the concept of molecular imaging. The future of molecular imaging lies in combining almost all contrast agents and radiotracers to specific ligands, such as membrane components of macrophages, platelets, oxidized low-density lipoproteins (LDL), and activated endothelia [80,81].

## Results

### FDG-PET Imaging

#### Relationship between FDG Uptake and Biological Activities

In 2002, Sakalihasan et al. conducted a pioneering study with the underlying rationale that association of AAA prone to rupture with inflammatory cell infiltrate could be detected by increased signaling on FDG PET [82]. All 10 of the 26 patients included had a short-term clinical event, whereas only 2 of the 16 remaining showed rapid growth, suggesting a possible association between increased FDG uptake in AAA and clinical outcomes. Since then, several studies have been launched, first to confirm the association between FDG uptake and the physiopathological events precluding AAA rupture. It has been widely shown that FDG uptake is associated with inflammatory and phagocytic cell infiltrates [56,83,84,85,86], proteolytic activity by matrix metalloproteases [83,87], and cellular and molecular signaling prefacing rupture [88]. However, there were arguments that FDG uptake is a non-specific [89] and uncommon [90,91] figure in AAAs since they are characterized by a cell-density decrease [86].

#### FDG Uptake and AAA Growth and Rupture Prediction

**Figure 6:**
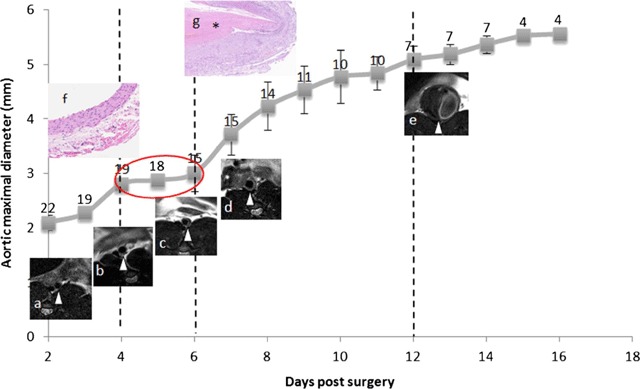
Post-elastase infusion (surgical) diameter growth curve of the aorta. Each point represents the number of rats, mean abdominal aortic diameter, and standard deviation. Images (a–e) are time-line inserts of selected transverse MRI. The curve is characterized by four different phases separated by vertical dotted lines. There is a short central quiescent phase (red circle) in between days three to six post-surgery where there is a macroscopic ILT (b). This phase is surrounded by two growth phases, the first of which is characterized by a subtle wall thickening and the remaining by progressive thickening and stratification of the ILT. Images (f–g) are inserts of ×20 magnification hematoxylin-eosin histological views of the normal aortic wall (f) and the aneurismal wall after appearance of the ILT (g, asterisk), showing inflammatory infiltrates. Abbreviations as in the text (adapted from Nchimi et al. [60]).

**Figure 7:**
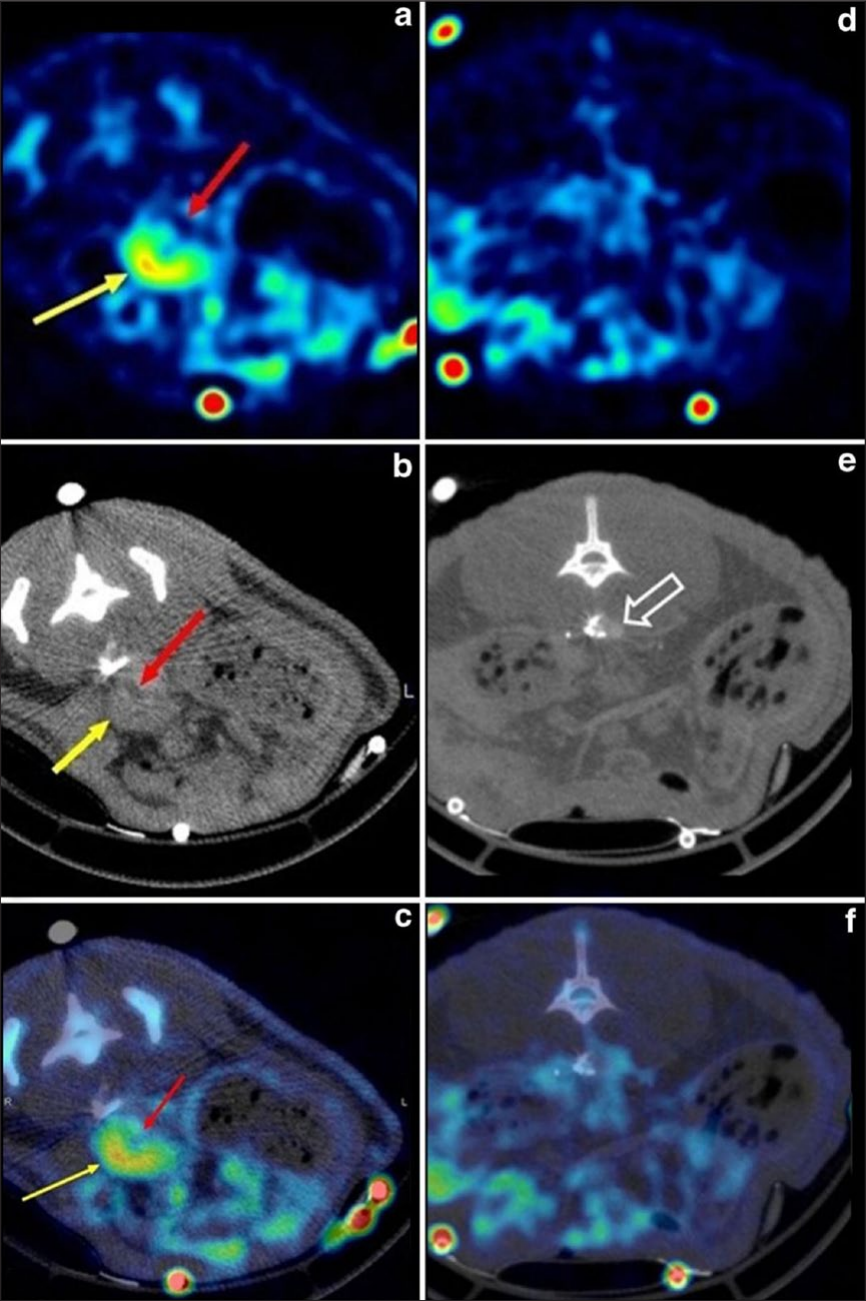
ILT positive (left panel) and ILT negative (right panel) prone transverse FDG PET (**A** and **D**), CT (**B** and **E**), and fused PET-CT (**C** and **E**) images of the aorta in rats, respectively, 13 and 5 days after infusion of elastase. On the left panel, the aorta (arrow) is largely dilated. The ILT is seen as a ventral thickening of the aortic wall containing two layers of different densities on CT. The luminal part of the ILT has a low density and exhibits low FDG uptake (red arrows), while the external part of the thrombus has a higher CT density and exhibits stronger FDG uptake (yellow arrows). On the right panel, the aorta (open arrow) is undilated, and neither intraluminal thrombus nor increased FDG uptake are seen. Abbreviations as in the text (adapted from Nchimi et al. [60]).

**Figure 8:**
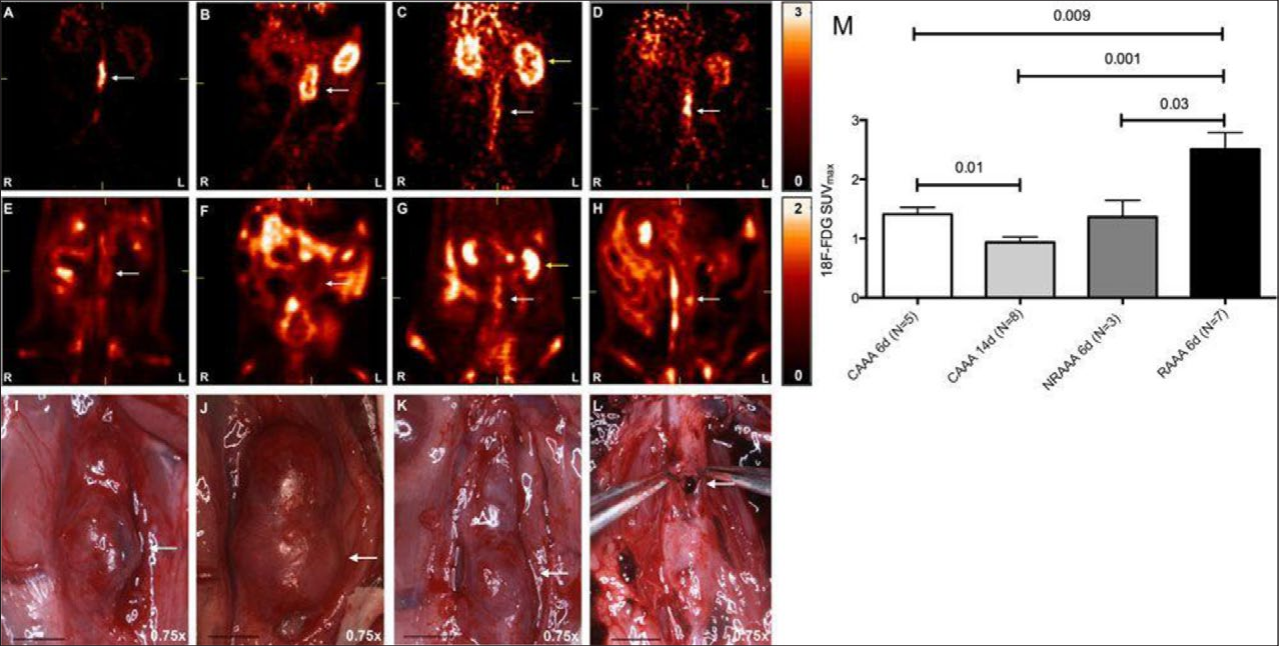
Micro-PET FDG uptake maps demonstrating increased focal uptake at the site of ultimate AAA rupture. Early phase represents the first 90 seconds of a 90-minute micro-PET scan, and late phase represents the last 30 minutes. **(A–D)** are coronal cuts for early phase images in control AAA at 6 days **(A)** and 14 days **(B)** after infusion of elastase, non-ruptured AAA **(C)** and ruptured **(D)** AAA 6 days after infusion of elastase, then rupture induction by daily subcutaneous β-aminopropionitrile. **(E–H)** are coronal cuts for late-phase images in the same animals, showing diffuse FDG uptake, with decreased uptake in the left anterolateral AAA wall, in all non-ruptured AAAs and focal FDG uptake in the left lateral wall of the ruptured AAA. **(I–L)** represent harvest photographs for the respective animals, magnification 0.75×, scale bar 5 mm. The rupture site correlated with the focal 18F-FDG uptake noted in image **(H)**. Yellow arrows identify the left kidney. Abbreviations as in the text (adapted from English et al. [92]).

**Figure 9:**
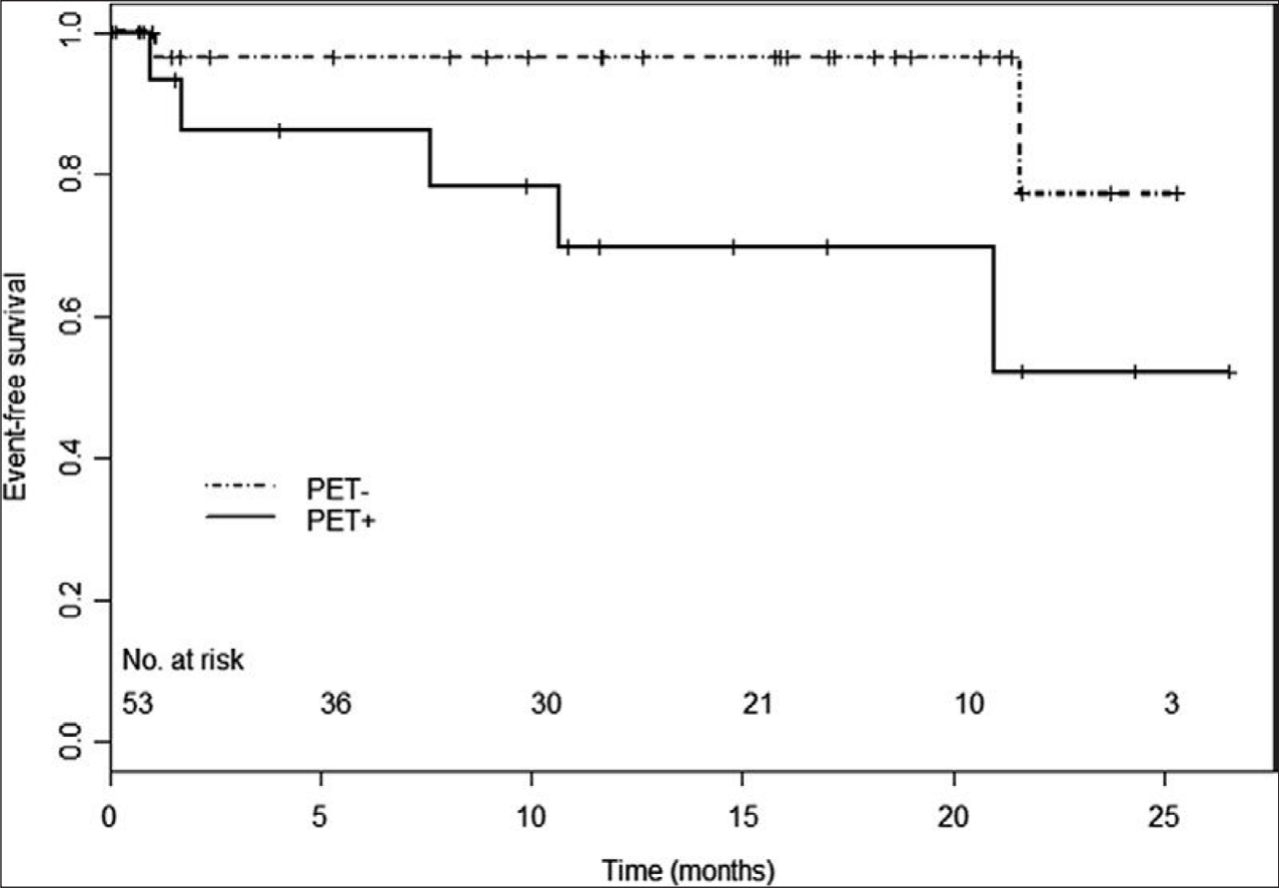
Two-year event-free survival curves in PET-positive and PET-negative patients according to increased FDG uptake. Abbreviations as in the text (adapted from Nchimi et al. [94]).

Animal models of AAA support the link between FDG uptake and both growing size and biological activities [60] (Figures 6 and 7). English et al. used rats that were exposed to intra-aortic porcine pancreatic elastase and underwent daily subcutaneous injection of β-aminopropionitrile (an inducer of AAA rupture) in one group and saline in the remaining rats [92]. All the rats underwent sequential FDG micro-PET examinations, and rupture was monitored by radiotelemetry. FDG uptake associated with inflammation in the AAA wall and focally increased at future sites of rupture in comparison to the control rats (Figure 8). To date, there have been few studies evaluating aneurysm outcomes relative to FDG uptake in humans. In the studies reporting rupture as the endpoint in patients with FDG PET/CT, the site of rupture almost always spatially co-localize with an area of uptake [82,93]. Studying 53 patients with aortic aneurysms, Nchimi et al. found that a higher rate of a composite outcome (rupture, dissection, or growth > 1 cm) occurred in patients within two years after a PET/CT examination showing a visually increased 18F-FDG uptake [94] (Figure 9). This replicates smaller series reported earlier by Sakalihasan et al. observing 26 patients [82] and Xu et al. observing 5 patients [93].

When the outcome with regards to the FDG uptake is the aneurysm growth rate, the results seem to be more conflicting, according to a recent meta-analysis [95]. Kotze et al. found no significant correlation between FDG uptake and AAA growth [85] in 14 AAAs under surveillance by using regular ultrasound. A study performed later by the same group in 25 patients similarly confirmed a negative correlation between FDG uptake and ultrasound expansion one year later [96]. Morel et al. set up a different study by evaluating 39 patients with medically treated AAA who underwent an FDG PET/CT at baseline and nine months later. The 9 patients showing a significant increase in maximal diameter (≥ 2.5 mm) during the nine-month period had (i) a lower SUVmax in the AAA at baseline than the other patients, (ii) a higher variability of SUVmax between the two FDG PET/CT scans, and (iii) similar values of SUVmax compared to the other patients at the second observation. These results suggest a lower level of FDG uptake before a growth phase and, more interestingly, a pattern of cyclic metabolic changes in the AAA wall [97].

#### Limitations

One of the main limitations of FDG PET is technical. Partial volume effect observed in small targets, such as the AAA wall, and spillover of lumen activity may alter the measures’ accuracy. Results and uptake quantification could be more accurate by implementing volume effect correction. FDG has the inconvenience of being nonspecific. Therefore, FDG uptake would not necessarily be associated with a deleterious outcome, depending on the cells or cell subtypes present in the aneurysm. For example, there are two antagonist subtypes of macrophages within vascular lesions that could not be distinguished by FDG PET: one pro-atherogenic and pro-inflammatory type, called M1, and the opposite, called M2 [98]. It nevertheless makes sense that FGD uptake tracks inflammation, but the counterhypothesis that inflammatory activity in the aortic wall would be the consequence of expansion rather than the cause has never been tested.

Another part of the conflicting results with the use of FDG PET to predict AAA-related outcomes is attributable to the study groups that are small, mostly including large aneurysms and some descending thoracic aneurysms [93,94]. Indeed, there is no evidence that FDG uptake is similar in large versus small aneurysms or in thoracic versus abdominal aneurysms. In addition, there is the fact that the aneurysms near the surgical thresholds obviously prevent long-term follow-ups. More importantly, these studies are based on time-point observations, which probably tell little about the dynamic nature of the ongoing processes in the aortic wall under the influence of other risk factors, such as increases in blood pressure, infection, and so on. Considering hypothesis of a cyclic pattern of metabolic changes in the AAA wall, a significantly increased FDG uptake may nevertheless prove to have a high positive predictive value and prompt AAA surgery repair after due confirmation by further research overcoming the current limitations.

### Future Imaging Tools to Assess the Risk of Rupture in AAA

**Figure 10:**
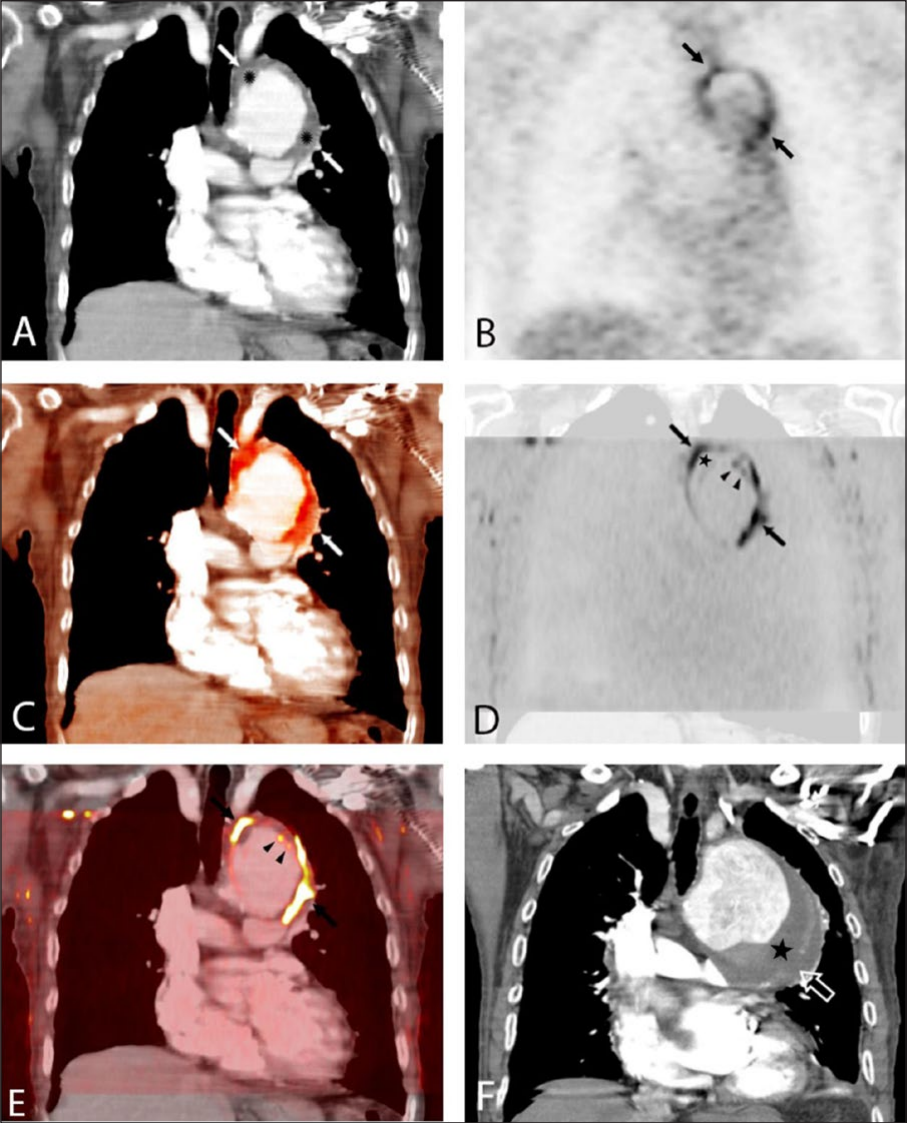
**(A)** Coronal reformatted contrast-enhanced computed tomography (CT) of the chest demonstrates a large aortic arch aneurysm with an intraluminal thrombus (ILT) (stars). **(B)** 18F-fluorodeoxyglucose positron emission tomography (FDG-PET) and **(C)** color intensity maps fusion with CT showed FDG uptake on the aneurysm wall (arrows, **D**). Coronal reformats of transverse diffusion-weighted magnetic resonance images with a diffusion factor value of 800 sec/mm² were fused to CT images in a similar plane **(E)** and showed restricted diffusion on the aneurysm wall (arrows) but differed from FDG-PET by increased signaling on the luminal surface of the ILT (arrowheads). The patient died three months later. **(F)** Admission CT showed aortic enlargement and rupture upon thrombus-covered aneurysm wall (open arrow) (from Nchimi et al. [100]).

Future imaging tools for assessing the risk of rupture include the progress of current clinical imaging techniques and widespread use of other technical concepts. Among the promises raised from novel assessment of routinely used techniques is textural image analysis. In a recent study involving 50 patients with AAA, among whom 40 underwent ultrasound follow-up for one year after initial PET/CT, it was reported that the signal heterogeneity of the aneurismal wall components on CT correlated well with FDG uptake, thus reflecting the metabolic activity [99]. Further, CT signal heterogeneity and FDG uptake were strong predictors for expansion. MRI also offers many opportunities for rupture risk assessment in AAAs, one of which is DWI. The similarity between DWI and FDG PET in aortic aneurysm has so far been reported only once in a patient with an aortic arch aneurysm (100) (Figure 10) [100]. Tissue perfusion is another prospect of MRI, aimed at evaluating periaortic neoangiogenesis as a marker of instability, as shown in preliminary reports [101,102].

New contrast agents and tracers with organ or physiological process-specific affinity are highly suitable. The use of iron oxide particles that have affinity for the reticulo-endothelial system has shown promises in evaluating macrophage adsorption at the luminal surface of the ILT [103] and outcomes in AAAs [104] before these contrast agents became commercially unavailable. A recent comparison of FDG and iron oxide uptake, on PET and MRI, respectively, in 15 patients with AAA, suggests the targets of the two techniques may be different cellular groups [105]. The future of metabolic and molecular imaging therefore lies in the combination of contrast agents and radiotracers to specific ligands, such as membrane components of macrophages, platelets, activated endothelia, and oxidized low-density lipoproteins [80,81].
